# Predictive Utility of EROA/LVEDV Ratio in Mitraclip Outcomes: Retrospective Multicenter Cohort Study

**DOI:** 10.3390/medicina62040795

**Published:** 2026-04-21

**Authors:** Vivek Joseph Varughese, Chandler Richardson, James Pollock, Patryk Czyzewski, Ashley Lyons, Hata Mujadzic, Deborah M. Hurley, Michael Cryer, Sunil V. Rao, Akshay Kumar

**Affiliations:** 1 Department of Medicine, Prisma Health, University of South Carolina School of Medicine, Columbia, SC 29203, USA; chandler.richardson@prismahealth.org (C.R.); james.pollock@prismahealth.org (J.P.); patryk.czyzewski@prismahealth.org (P.C.); ashley.mason@prismahealth.org (A.L.);; 2 Department of Cardiology, Prisma Health, University of South Carolina School of Medicine, Columbia, SC 29203, USA; hata.mujadzic@prismahealth.org (H.M.); michael.cryer3@prismahealth.org (M.C.); 3 Department of Medicine, New York University Grossman School of Medicine, New York, NY 10016, USA; sunil.rao@nyulangone.org (S.V.R.); drakshay82@gmail.com (A.K.)

**Keywords:** Functional Mitral Regurgitation, transcatheter edge-to-edge repair, Mitraclip

## Abstract

*Background*: The effective regurgitant orifice area to left ventricular end-diastolic volume (EROA/LVEDV) ratio has been proposed to distinguish proportionate from disproportionate functional mitral regurgitation and to guide patient selection for transcatheter edge-to-edge repair (TEER). *Methods*: We conducted a multicenter, retrospective cohort study of 221 patients undergoing TEER with the Mitraclip system. Preprocedural echocardiographic parameters, including EROA, LVEDV, diastolic indices, and chamber volumes, were systematically collected. The primary outcome indicative of symptom worsening was defined as Heart Failure Hospitalizations (HFH) requiring IV diuresis/death in the one year following clip placement. Association of the preprocedural EROA/LVEDV ratio and symptom worsening was assessed using multivariate regression models and ROC-AUC. *Results*: In the one-year follow-up, 87 patients (39.36%) had symptom worsening. In the multivariate regression analysis, preprocedural EROA/LVEDV ratio was associated with symptom worsening at one year (OR: 0.95 (0.92–0.97, *p* value < 0.01). In the ROC model, the pre-procedural EROA/LVEDV ratio had an AUC value of 0.74 (0.69–0.83), with a moderate value for predicting symptom worsening at one year. *Conclusions*: Results of the study proved that a lower pre-procedural EROA/LVEDV ratio had a significant association with symptom worsening, with the ratio proving to have a moderate value for predicting symptom worsening/death at one year.

## 1. Introduction

Since its initial approval, the use of Mitraclip has expanded rapidly, particularly following the FDA’s expanded indication for Functional Mitral Regurgitation (FMR) [1]. Current ACC/AHA guidelines endorse transcatheter edge-to-edge repair (TEER) with the Mitraclip device as a Class IIa recommendation for patients with chronic severe FMR who remain symptomatic despite maximally tolerated guideline-directed medical therapy (GDMT), provided they meet anatomical and clinical criteria suitable for the procedure [2]. This recommendation is largely informed by the findings of the COAPT trial, while another landmark trial, the MITRA-FR, did not demonstrate a significant reduction in All-Cause Mortality (ACM) or Heart Failure Hospitalizations (HFH) with Mitraclip compared to medical therapy alone, despite enrolling patients with severe FMR [3,4]. Insights have been proposed by Packer and Grayburn, who articulated a conceptual framework distinguishing “proportionate” from “disproportionate” FMR—based on whether the effective regurgitant orifice area (EROA) is proportional to left ventricular end-diastolic volume (LVEDV)—to explain the divergent results between COAPT and MITRA-FR trials [5,6]. These findings underscore the potential value of the EROA/LVEDV ratio as a physiologic marker aiding clinicians to stratify patients with proportionate Mitral Regurgitation (MR) who are more likely to respond to neurohormonal strategies and other advanced heart failure therapies, compared to disproportionate FMR who are more likely to benefit from mitral valve interventions [7,8,9].

Our study aimed to evaluate the association between preprocedural EROA/LVEDV ratio and one-year clinical outcomes following transcatheter edge-to-edge repair (TEER) using the Mitraclip device. We hypothesized that patients with a lower degree of regurgitation relative to left ventricular size would be a predictor of worsening clinical status in one year following Mitraclip placement

## 2. Materials and Methods

The study was designed in a retrospective cohort format. Patients who underwent mTEER using Mitraclip at Prisma Health Richland Hospital, Prisma Health Baptist Hospital and Prisma Health Greenville campus (South Carolina, USA) between January of 2021 and March of 2024 were selected for the study.

Inclusion Criteria: Patients aged ≥ 18 years, who underwent successful Mitraclip placement for FMR, with a 3+/4+ severity of mitral regurgitation, were included.

Exclusion Criteria: Patients undergoing Mitraclip placement for Primary/Degenerative Mitral Regurgitation were excluded from the study. Patients with comorbid mitral stenosis were excluded from the study.

After careful manual chart review of 326 patients, 221 patients were included in the study. 36 patients were excluded due to unsuccessful procedure/clip deployment. 43 patients were excluded due to primary/degenerative MR being the indication, 8 patients had documented Mitral stenosis, 18 patients were excluded because of combined degenerative and functional MR being the indication for the procedure, and 10 patients were excluded as no follow-up data was available for chart review. One-year outcomes were analyzed for the selected patients according to the criteria defined by the Mitral Valve Research Consortium on efficacy and safety endpoints. All patients included in the analysis had documented successful implantation, defined by the absence of procedural mortality, successful access, delivery, and retrieval of the device delivery system, successful deployment and correct positioning of the first intended device and freedom from emergency surgery or reintervention related to the device or access procedure.

As per the recommendations of the mitral valve Academic Research Consortium, the primary endpoint was symptom worsening, defined by hospitalizations for heart failure/volume overload in the one year following mTEER placement, requiring IV diuretics in the one-year post Mitraclip placement.

Pre-procedural echocardiographic assessment was performed according to American College of Cardiology and American Society of Echocardiography guidelines. Effective regurgitant orifice area (EROA) was quantified using the proximal isovelocity surface area (PISA) method, and left ventricular end-diastolic volume (LVEDV) was calculated using the biplane method of disks summation (modified Simpson’s rule) from apical four- and two-chamber views, with manual tracing of the endocardial border at end-diastole, excluding papillary muscles and trabeculae from the LV cavity.

### Statistical Analysis

Descriptive statistics were used to summarize patient and clinical data. Initial evaluation of differences in patient and clinical data by group were assessed using Chi-squared or Fisher’s Exact Tests for categorical data, and Wilcoxon Rank Sum or Mood’s Median Tests for numeric data. Univariable logistic regression, followed by multivariable logistic regression (using backwards elimination), was used to obtain unadjusted and adjusted odds ratios (ORs) with 95 percent confidence intervals (CIs). Age, Sex, ACE/ARB/Mineralocorticoid use post-procedure, CKD history, use of Cardiac Resynchronization Therapy, pre-procedural E/A ratio, Pulmonary artery systolic pressure, and diagnosis of atrial fibrillation were accounted for as covariates in the regression analysis. Statistical significance was determined by 95% CIs or resulting *p*-values (*p* < 0.05) as appropriate. ROC/AUC models were based on significant results from the multivariable logistic regression analyses.

IRB Approval: Prisma Health IRB: [2216293-1] Outcomes for Mitral Clip Placement: Multi-Center Retrospective Cohort Analysis: Exempt category #4 (Informed consent waived).

## 3. Results

In the one-year follow-up, 87 patients (39.36%) had symptom worsening. Baseline demographic and echocardiographic data of the outcome groups are depicted in Table 1. Mean age and sex distribution were comparable among the outcome groups. Considering GDMT adherence, a significant difference was noted in the use of mineralocorticoid receptor antagonist among outcome groups. EROA as well as LVEDV were also different among outcome groups. Patients with symptom worsening had a significantly lower participation in cardiac rehabilitation post-procedure.

Results of the univariate regression analysis checking the association of clinical and echocardiographic parameters with the primary endpoint are depicted in Table 2.

In the univariate regression analysis, MRNA adherence, CKD history, and history of PCI were statistically different among the outcome groups. EROA, LVEDV, LVEDD, LVESD, E/A ratio and MV deceleration time were the echocardiographic parameters that were different among the outcome groups. In the univariate regression analysis, lower EROA/LVEDV was found to have a significant association with symptom worsening.

Analysis of the EROA/LVEDV ratio among symptom groups is depicted in Table 3.

Results of the Multivariate Regression Analysis assessing the association between preprocedural EROA/LVEDV ratio and primary endpoint/symptom worsening are depicted in Table 4.

Based on the multivariate regression analysis, a higher pre-procedural EROA/LVEDV ratio was associated with significantly lower odds of symptom worsening after Mitraclip placement (OR 0.95, 95% CI 0.92–0.97, *p* < 0.01). When scaled to a clinically meaningful increment, each 0.1 mm^2^/mL increase in the EROA/LVEDV ratio was associated with approximately a 0.5% reduction in the odds of symptom worsening. The narrow confidence interval and statistically significant *p*-value suggest a robust association.

**Figure 1:**
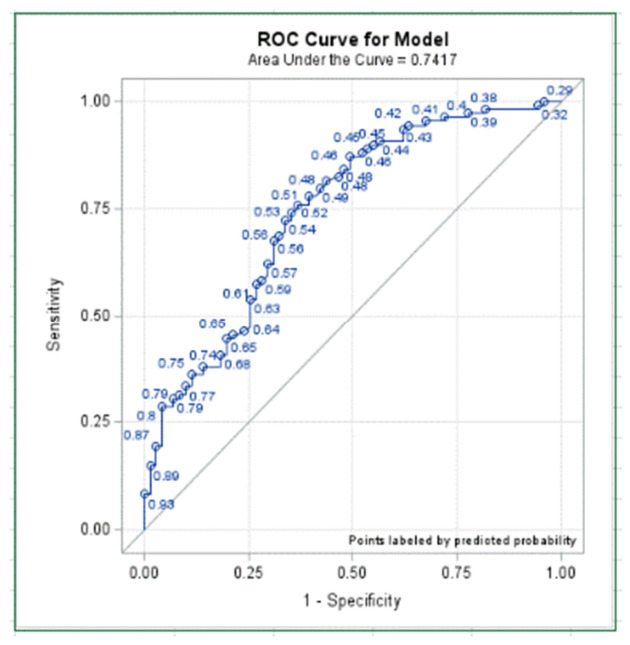
ROC curve model. ROC: Receiver Operating Characteristic.

ROC/AUC Analysis between EROA/LVEDV and symptom worsening was performed after accounting for pre-procedural E/A ratio and Mineralocorticoid Use (based on significance in the multivariate regression analysis). Results and the ROC/AUC are shown in Table 5 and Figure 1.

The ROC/AUC analysis demonstrated that the EROA/LVEDV ratio has good discriminatory ability for predicting symptom worsening after mTEER, with an area under the curve (AUC) of 0.74 (95% CI 0.69–0.83). This analysis was adjusted for ACE inhibitor/ARB/ARNI (sacubitril-valsartan) use and mineralocorticoid receptor antagonist use, variables identified as significant in the multivariate regression model. An AUC of 0.74 indicates that the EROA/LVEDV ratio correctly distinguishes between patients who will and will not experience symptom worsening approximately 74% of the time, which is considered acceptable to good diagnostic performance. The relatively narrow confidence interval (0.69–0.83) and standard error of 0.036 suggest reasonable precision in this estimate.

## 4. Discussion

Based on our study, a lower preprocedural EROA/LVEDV ratio (expressed in mm^2^/mL) had a significant association with symptom worsening at one year following MitraClip placement for FMR. Patients who reached the primary efficacy endpoint had a mean EROA of 52 mm^2^ (95% CI: 43–61 mm^2^) and a mean LVEDV of 74.56 mL (62.55–86.57), resulting in a mean EROA/LVEDV ratio of 0.37 mm^2^/mL (95% CI: 0.33–0.41). In contrast, those with symptom worsening had a mean EROA of 38 mm^2^ (95% CI: 32–44 mm^2^), mean LVEDV of 157 mL (95% CI: 142–166 mL), and a lower ratio of 0.22 mm^2^/mL (95% CI: 0.19–0.25). On multivariate regression analysis, EROA/LVEDV ratio was independently associated with symptom worsening (OR: 0.95, 95% CI:0.92–0.97 *p* < 0.0001). ROC analysis, with an AUC of 0.74 (95% CI: 0.69–0.83), aligns with a moderate predictive utility of the EROA/LVEDV ratio in identifying patients likely to have symptom worsening following Mitraclip placement.

The pathophysiologic rationale behind the EROA/LVEDV ratio lies in its ability to contextualize MR severity relative to left ventricular size, thereby distinguishing between different mechanistic subtypes of FMR. It accounts for scenarios in which localized valvular dysfunction, rather than global ventricular dilation, is the predominant driver of regurgitation. There is growing recognition that asymmetric papillary muscle dysfunction—often due to electrical or mechanical dyssynchrony—can result in malcoaptation of the mitral valve leaflets and significant MR, even in the presence of relatively preserved or modestly dilated LV chambers. This pattern is frequently observed in patients with left bundle branch block or posteromedial infarction, where asynchronous contraction leads to uneven tethering forces and displacement of one leaflet, producing disproportionately severe MR. Unlike classic FMR, which stems from symmetrical leaflet tethering due to diffuse LV enlargement, these cases mimic the mechanics of primary MR irrespective of the underlying LV dysfunction. In the subgroup analysis from the COAPT trial, which stratified patients with FMR based on EROA and left ventricular end-diastolic volume index (LVEDVi) to examine the relative benefits of TEER, patients with disproportionate MR, defined by a higher EROA-to-LVEDV ratio (e.g., EROA > 40 mm^2^ and LVEDVi ≤ 96 mL/m^2^, mean ratio 0.37 mm^2^/mL), derived the most substantial benefit from TEER, with a hazard ratio (HR) of 0.61 (95% CI: 0.33–1.12) for ACM and HFH at 12 months. In contrast, the only subgroup classified as having proportionate MR (EROA ≤ 30 mm^2^ and LVEDVi > 96 mL/m^2^, mean ratio 0.11–0.12 mm^2^/mL) showed no meaningful benefit from TEER, with a HR of 1.16 (95% CI: 0.73–1.84) [5]. Other subgroup analysis studies have further supported the conceptual framework of proportionate versus disproportionate FMR [10,11]. Further supporting the clinical relevance of the EROA/LVEDV ratio, a recent single-center study by Chiariello et al. demonstrated that patients with disproportionate mitral regurgitation (defined as EROA/LVEDV > 0.15 mm^2^/mL) had significantly fewer heart failure hospitalizations and better functional outcomes following mitral valve surgery, despite receiving similar operative interventions [12]. These findings reinforce the physiologic and prognostic utility of the EROA/LVEDV ratio and align closely with our study’s observation that lower ratios are independently associated with worsened symptomatic outcomes following transcatheter edge-to-edge repair. However, other analyses have questioned the predictive utility of this framework in broader populations. In the post hoc analysis of the COAPT trial by Lindenfeld et al., it was observed that although the EROA/LVEDV ratio was initially associated with favorable outcomes, this association diminished with longer follow-up, limiting its long-term discriminative value [13]. Subsequent meta-analyses as well as subgroup analyses have also questioned the predictive utility of the score [14]. While the EROA/LVEDV ratio offers valuable mechanistic insight, it may need to be interpreted alongside additional clinical, anatomical, and hemodynamic parameters to guide optimal patient selection for TEER. The delayed clinical benefit in an MITRA-FR–like population underscores the possibility that temporal dynamics in treatment response may also influence outcomes [11,12].

Several potential explanations may underline the inconsistent findings across real-world and randomized studies evaluating the EROA/LVEDV ratio. First, optimization of guideline-directed medical therapy (GDMT) differed substantially between trials. COAPT incorporated a Heart Failure Committee to ensure stringent adherence both pre- and post-enrollment, whereas MITRA-FR lacked such oversight, which may have introduced variability in baseline management and attenuated procedural benefit [15]. Another potential aspect of consideration is whether the defined end points of HFH and ACM correlate with functional outcomes, as real-world studies from the EuroSMR registry [14] proved that although LV dominant MR group had significantly higher HFH and ACM, functional outcomes like improvement in the baseline Kansas City Cardiomyopathy Questionnaire (KCCQ) scores and 6 Minute Walk test were comparable among outcome groups. Additionally, Atrial Fibrillation (AF) prevalence was nearly twice as high in COAPT compared to MITRA-FR (58% vs. 30%), potentially accounting for elevated natriuretic peptide levels in COAPT and influencing patient selection and outcomes [3]. The presence or absence of cardiac resynchronization therapy (CRT) may also play a role, as dyssynchrony can exacerbate asymmetric papillary muscle dysfunction—a pathophysiologic substrate of disproportionate MR—and CRT can acutely improve mitral valve geometry and reduce regurgitation independent of LV remodeling. Chronic kidney disease (CKD), another relevant confounder, has been associated with poorer long-term Mitraclip outcomes, potentially diminishing the predictive utility of anatomical ratios such as EROA/LVEDV in this subpopulation [16]. Baseline functional status is also critical: worse NYHA class or lower Kansas City Cardiomyopathy Questionnaire (KCCQ) scores have been associated with diminished post-procedural improvement and may independently influence perceived benefit despite anatomical severity [17,18]. Lastly, pre-procedural diastolic dysfunction, an often-overlooked parameter in the current patient selection criteria, may confound symptom trajectories following Mitraclip; in a multicenter real-world cohort, advanced diastolic dysfunction was significantly associated with poorer one-year symptomatic response, regardless of regurgitant burden [19]. A key strength of our analysis lies in its comprehensive multivariate adjustment of these clinical and echocardiographic factors known to influence outcomes in FMR. These included atrial fibrillation, chronic kidney disease, cardiac resynchronization therapy, and adherence to guideline-directed medical therapy—further stratified by ACE/ARB and mineralocorticoid receptor antagonist use. We also accounted for pulmonary artery systolic pressure and diastolic function markers such as the E/A ratio in the multivariate regression analysis.

While the EROA/LVEDV ratio provides a useful physiological framework for identifying disproportionate functional mitral regurgitation (FMR), its predictive value may be enhanced by incorporating regurgitant volume (RV). In the study by Gaasch et al., patients in the COAPT and MITRA-FR trials demonstrated similar RV/LVEDV ratios (0.15 vs. 0.18), suggesting that EROA-based proportionality alone may not fully capture MR severity or clinical benefit [20]. Likewise, Grayburn et al. highlighted that EROA reflects instantaneous flow, whereas RV better represents the cumulative regurgitant burden over systole and is influenced by ventricular geometry and loading conditions [21]. Consequently, patients with similar EROA/LVEDV ratios may have markedly different hemodynamic profiles if the RV differs. Additionally, both EROA and RV measurements are susceptible to geometric assumptions and technical variability—particularly in atrial fibrillation or eccentric jets—as noted by Uretsky et al., potentially limiting real-world applicability [22]. Vena contracta (VC) assessment, especially the three-dimensional vena contracta area (3D-VCA), has emerged as a more reproducible metric that directly quantifies the anatomic regurgitant orifice, with less dependence on hemodynamic conditions. Smaller post-procedural 3D-VCA has been associated with improved 1-year survival and reduced heart failure hospitalizations, supporting its prognostic utility [23,24]. These considerations are particularly relevant in patients with advanced heart failure and significant ventricular remodeling, where persistent symptoms despite optimal therapy may warrant earlier evaluation for durable mechanical support, including LVAD or heart transplantation [25,26].

### Limitations of the Study

This study was designed as an observational retrospective cohort model and did not contain a control group. A potential patient selection bias cannot be excluded, but the indications for TEER were approved as per the standardized AHA/ACC criteria. Another limitation of our study is the lack of RV and VC data, as these were not routinely recorded across the centers. Additionally, the follow-up period was limited to 12 months; thus, it remains unclear whether the predictive utility of the EROA/LVEDV ratio would persist over longer durations. While GDMT adherence and resynchronization were used in the multivariate regression analysis, the intensity and duration of treatment with specific GDMT/Resynchronization therapies could not be assessed in the study. Hence the treatment-related confounding related to GDMT use and mechanical dysynchrony due to LV failure could not be accounted for.

## 5. Conclusions

The concept of disproportionate regurgitation offers valuable physiologic insight but requires further refinement through integration of broader clinical and imaging factors. Continued multicenter investigations are needed to better define patient selection criteria and optimize TEER outcomes.

## Figures and Tables

**Table 1:** Baseline patient characteristics.

	Improved/Stable (n = 134)	Worsened (n = 87)	*p*-Value
**Mean Age**	**72.32 (70.17–74.56)**	**70.51 (67.71–73.34)**	0.367
**Sex, n (%)**	Male: 84 (63.21)	Male: 58 (68.23)	
	Female: 49 (36.84)	Female: 27 (31.86)	
**Race, n (%)**			
White	91 (68.41)	40 (47.15)	0.004
Black	39 (29.34)	43 (50.67)	
Hispanic	2 (1.51)	1 (1.20)	
Other	1 (0.81)	1 (1.24)	
**ACE/ARB/Entresto use ** **(post procedure)**	81 (62.32)	50 (61.01)	0.846
**Beta-blocker use ** **(post procedure)**	110 (84.02)	75 (92.61)	0.067
**MRA use ** **(post procedure)**	57 (43.85)	52 (63.46)	0.006
**SGLT2 inhibitor use ** **(post procedure)**	72 (56.73)	45 (67.26)	0.156
**Rehabilitation participation**	42 (33.12)	6 (7.45)	<0.001
**Atrial fibrillation**	90 (68.74)	62 (75.68)	0.278
**NYHA Class ** **(pre-procedure)**	1 (0.81)	1 (1.30)	0.279
	12 (9.20)	13 (16.31)	
	112 (86.22)	61 (76.35)	
	5 (3.84)	5 (6.32)	
**CKD history**	71 (53.42)	57 (67.15)	0.046
**CKD Stage, n (%)**			0.349
**Stage 1**	7 (7.91)	2 (3.30)	
**Stage 2**	23 (25.81)	9 (15.01)	
**Stage 3**	42 (47.27)	36 (60.01)	
**Stage 4**	10 (11.22)	8 (13.35)	
**Stage 5**	7 (7.93)	5 (8.35)	
**COPD**	55 (42.01)	35 (43.85)	0.801
**MI history**	57 (44.25)	24 (30.01)	0.041
**Pulmonary hypertension**	83 (62.46)	62 (74.75)	0.061
**Cardiac Resynchronization Therapy**	54 (41.53)	30 (37.01)	0.516
**Severe Tricuspid regurgitation**	13 (10.27)	5 (6.33)	0.605
**2 or more clips used**	127 (97.75)	72 (96.01)	0.671
**EROA (cm^2^) ** **(pre-procedural)**	0.5 ± 0.21	0.4 ± 0.34	<0.01
**LAVi ** **(pre-procedural)**	57.6 ± 24.71	60.8 ± 35.93	0.737
**LVEDV (mL) ** **(pre-procedural)**	74.56 ± 22	157.2 ± 29	0.005
**Mitral Valve Gradient (mm Hg) ** **(pre-procedural)**	2.21 ± 1.52	1.96 ± 0.94	0.700
**Mitral Valve Gradient (mm Hg) ** **(post-procedural)**	3.17 ± 1.21	2.94 ± 0.12	0.082
**RVSP (mm Hg) ** **(pre-procedural)**	46.32 ± 27.91	48.77 ± 12.35	0.019

ACE/ARB: Angiotensin Converting Enzyme inhibitor/Angiotensin Receptor Blocker, SGLT2i: Sodium Glucose co-Transporter 2 inhibitor, MRA: Mineralocorticoid Receptor Antagonist, CRT: Cardiac Resynchronization Therapy, NYHA: New York Heart Association, CKD: Chronic Kidney Disease, MI: Myocardial Infarction, COPD: Chronic Obstructive Pulmonary Disease, EROA: Effective Regurgitant Orifice Area, LVEDV: Left Ventricular End Diastolic Volume, LAVi: Left Atrial Volume index, RVSP: Right Ventricular Systolic Pressure.

**Table 2:** Univariate Regression analysis .

	OR (95% CI)	*p*-Value
ACEi/ARB/ENTRESTO adherence	0.94 (0.53–1.67)	0.846
Beta Blocker adherence	2.38 (0.92–6.25)	0.074
MRA adherence	2.22 (1.27–3.85)	0.006
Variable	1.56 (0.84–2.94)	0.158
CRT	0.83 (0.47–1.47)	0.516
AF History	1.41 (0.76–2.63)	0.279
CKD History	1.79 (1.01–3.13)	0.047
COPD History	1.08 (0.61–1.89)	0.801
ESRD on HD	1.08 (0.29–4.00)	0.914
History of PCI	0.54 (0.30–0.98)	0.042
Pulmonary Hypertension (>60 mm Hg)	1.49 (0.61–3.70)	0.376
MR EROA (mm^2^)	0.09 (0.02–0.44)	0.003
LVEDV (mL)	1.01 (1.00–1.01)	0.002
LAVi (mL/m^2^)	1.00 (0.99–1.01)	0.522
LA Diameter (cm)	1.04 (0.93–1.18)	0.494
LVEDD (cm)	1.56 (1.18–2.08)	0.002
RVSP (mm Hg)	1.00 (0.99–1.02)	0.512
LVESD (cm)	1.27 (1.01–1.59)	0.036
E/A Ratio	1.39 (1.03–1.89)	0.030
Transmitral Gradient (mm Hg)	0.86 (0.67–1.11)	0.240
MV Deceleration Time (ms)	0.99 (0.99–1.00)	0.005
EROA/LVEDV ratio (mm^2^/mL)	0.94 (0.93–0.97)	<0.001

ACEi/ARB: Angiotensin Converting Enzyme inhibitor/Angiotensin Receptor Blocker, MRA: Mineralocorticoid Receptor Antagonist, AF: Atrial Fibrillation, CRT: Cardiac Resynchronization Therapy, CKD: Chronic Kidney Disease, COPD: Chronic Obstructive Pulmonary Disease, ESRD: End Stage Renal Disease, PCI: Percutaneous Coronary Intervention, EROA: Effective Regurgitant Orifice Area, LVEDV: Left Ventricular End Diastolic Volume, LAVi: Left Atrial Volume index, LVEDD: Left Ventricular End Diastolic Diameter, RVSP: Right Ventricular Systolic pressure, LVESD: Left Ventricular End Systolic Dimension, E/A: Mitral annular E/A velocity.

**Table 3:** Mean EROA/LVEDV ratio among symptom groups.

	Mean EROA/LVEDV (mm^2^/mL)	95% Confidence Interval *	*p* Value
Group 1 (n = 134)	0.37	0.33–0.41	<0.01
Group 2 (n = 87)	0.22	0.19–0.25	<0.01

EROA: Effective Regurgitant Orifice Area, LVEDV: Left Ventricular End Diastolic Volume, *: Using Wilcoxon Rank Sum test.

**Table 4:** Multivariate Regression Analysis comparing EROA/LVEDV with symptom worsening.

	Odds Ratio	95% Confidence Interval *	*p* Value
EROA/LVEDV (mm^2^/mL)	0.95	0.92–0.97	<0.01
MRA adherence	0.72	0.64–0.81	0.003
E/A ratio	0.62	0.44–0.88	<0.001

EROA: Effective Regurgitant Orifice Area, LVEDV: Left Ventricular End Diastolic Volume, MRA: Mineralocorticoid Receptor Antagonist, E/A: Mitral annular E/A velocity, *: Walds 95% confidence interval.

**Table 5:** ROC/AUC statistics comparing EROA/LVEDV ratio and symptom worsening.

ROC Association Statistics			
ROC Model	Mann–Whitney		
	Area	Standard Error	95% Wald Confidence Limits
	0.74	0.036	0.69–0.83

## Data Availability

Data obtained through manual chart review of patients (Deidentified).

## References

[1] Mack M., Carroll J.D., Thourani V., Vemulapalli S., Squiers J., Manandhar P., Deeb G.M., Batchelor W., Herrmann H.C., Cohen D.J. (2021). Transcatheter mitral valve therapy in the United States: A report from the STS-ACC TVT Registry. J. Am. Coll. Cardiol..

[2] Otto C.M., Nishimura R.A., Bonow R.O., Carabello B.A., Erwin J.P., Gentile F., Jneid H., Krieger E.V., Mack M., McLeod C. (2021). 2020 ACC/AHA guideline for the management of patients with valvular heart disease. J. Am. Coll. Cardiol..

[3] Stone G.W., Lindenfeld J., Abraham W.T., Kar S., Lim D.S., Mishell J.M., Whisenant B., Grayburn P.A., Rinaldi M., Kapadia S.R. (2018). Transcatheter mitral-valve repair in patients with heart failure. N. Engl. J. Med..

[4] Obadia J.-F., Messika-Zeitoun D., Leurent G., Iung B., Bonnet G., Piriou N., Lefèvre T., Piot C., Rouleau F., Carrié D. (2018). Percutaneous repair or medical treatment for secondary mitral regurgitation. N. Engl. J. Med..

[5] Packer M., Grayburn P.A. (2020). New evidence supporting a novel conceptual framework for distinguishing proportionate and disproportionate functional mitral regurgitation. JAMA Cardiol..

[6] Grayburn P.A., Sannino A., Packer M. (2019). Proportionate and disproportionate functional mitral regurgitation: A new conceptual framework that reconciles the results of the MITRA-FR and COAPT trials. JACC Cardiovasc. Imaging.

[7] Koell B., Orban M., Weimann J., Kassar M., Karam N., Neuss M., Petrescu A., Iliadis C., Unterhuber M., Adamo M. (2021). Outcomes Stratified by Adapted Inclusion Criteria After Mitral Edge-to-Edge Repair. J. Am. Coll. Cardiol..

[8] Ypenburg C., Lancellotti P., Tops L.F., Bleeker G.B., Holman E.R., Piérard L.A., Schalij M.J., Bax J.J. (2007). Acute effects of initiation and withdrawal of cardiac resynchronization therapy on papillary muscle dyssynchrony and mitral regurgitation. J. Am. Coll. Cardiol..

[9] Packer M., Grayburn P.A. (2019). Contrasting effects of pharmacological, procedural and surgical interventions on proportionate and disproportionate functional mitral regurgitation in chronic heart failure. Circulation.

[10] Orban M., Karam N., Lubos E., Kalbacher D., Braun D., Deseive S., Neuss M., Butter C., Praz F., Kassar M. (2021). Impact of Proportionality of Secondary Mitral Regurgitation on Outcome After Transcatheter Mitral Valve Repair. JACC Cardiovasc. Imaging.

[11] Lindenfeld J., Abraham W.T., Grayburn P.A., Kar S., Asch F.M., Lim D.S., Nie H., Singhal P., Sundareswaran K.S., Weissman N.J. (2021). Association of effective regurgitant orifice area to left ventricular end-diastolic volume ratio with transcatheter mitral valve repair outcomes: A secondary analysis of the COAPT trial. JAMA Cardiol..

[12] Chiariello G.A., Di Mauro M., Villa E., Bruno P., Mazza A., Pavone N., Nesta M., Marcolini A., Panzera R., Armonia A. (2025). Disproportionate vs. Proportionate Secondary Mitral Regurgitation: A Long-Term Pilot Analysis After Mitral Valve Surgery. J. Clin. Med..

[13] Anker M.S., Porthun J., Bonnet G., Schulze P.C., Rassaf T., Landmesser U. (2024). Percutaneous Transcatheter Edge-to-Edge Repair for Functional Mitral Regurgitation in Heart Failure: A Meta-Analysis of 3 Randomized Controlled Trials. J. Am. Coll. Cardiol..

[14] Senni M., Adamo M., Metra M., Alfieri O., Vahanian A. (2019). Treatment of functional mitral regurgitation in chronic heart failure: Can we get a ‘proof of concept’ from the MITRA-FR and COAPT trials?. Eur. J. Heart Fail..

[15] Sisinni A., Munafò A., Pivato C.A., Adamo M., Taramasso M., Scotti A., Parlati A.L., Italia L., Voci D., Buzzatti N. (2022). Effect of chronic kidney disease on 5-year outcomes in patients with heart failure and secondary mitral regurgitation undergoing MitraClip. Am. J. Cardiol..

[16] Giustino G., Lindenfeld J., Abraham W.T., Kar S., Lim D.S., Grayburn P.A., Kapadia S.R., Cohen D.J., Kotinaduwa L.N., Weissman N.J. (2020). NYHA functional class and clinical outcomes in the COAPT trial. JACC Cardiovasc. Interv..

[17] Arnold S.V., Li Z., Vemulapalli S., Baron S.J., Mack M.J., Kosinski A.S., Reynolds M.R., Hermiller J.B., Rumsfeld J.S., Cohen D.J. (2018). Association of Transcatheter Mitral Valve Repair with Quality of Life Outcomes at 30 Days and 1 Year: Analysis of the Transcatheter Valve Therapy Registry. JAMA Cardiol..

[18] Varughese V.J., Richardson C., Pollock J., Czyzewski P., Mujadzic H., Cryer M. (2025). Diastology and MitraClip outcomes: A multicenter real-world evidence study. Medicina.

[19] Gaasch W.H., Aurigemma G.P., Meyer T.E. (2020). An appraisal of the association of clinical outcomes with regurgitant volume relative to end-diastolic volume in secondary mitral regurgitation. JAMA Cardiol..

[20] Grayburn P.A., Weissman N.J., Zamorano J.L. (2012). Quantitation of mitral regurgitation. Circulation.

[21] Uretsky S., Gillam L., Lang R., Chaudhry F.A., Argulian E., Supariwala A., Gurram S., Jain K., Subero M., Jang J.J. (2015). Discordance between echocardiography and MRI in the assessment of mitral regurgitation severity: A prospective multicenter trial. J. Am. Coll. Cardiol..

[22] Rottländer D., Sondermann N., Taramasso M., Bufe A., Seyfarth M., von Bardeleben R.S., Beucher H., Ouarrak T., Schneider S., Boekstegers P. (2025). Intraprocedural 3D vena contracta area predicts 1-year mortality following mitral transcatheter edge-to-edge repair: Results from the MITRA-PRO registry. Clin. Res. Cardiol..

[23] Alessandrini H., Kreidel F., Schlüter M., Frerker C., Schmidt T., Thielsen T., Schäfer U., Kuck K.H. (2017). Prognostic implication of post-MitraClip vena contracta area in functional mitral regurgitation. EuroIntervention.

[24] Avenatti E., Mackensen G.B., El-Tallawi K.C., Reisman M., Gruye L., Barker C.M., Little S.H. (2019). Diagnostic Value of 3-Dimensional Vena Contracta Area for the Quantification of Residual Mitral Regurgitation After MitraClip Procedure. JACC Cardiovasc. Interv..

[25] Godino C., Munafò A., Scotti A., Estévez-Loureiro R., Portolés Hernández A., Arzamendi D., Fernández Peregrina E., Taramasso M., Fam N.P., Ho E.C. (2020). MitraClip in secondary mitral regurgitation as a bridge to heart transplantation: 1-year outcomes from the International MitraBridge Registry. J. Heart Lung Transplant..

[26] Mangieri A., Melillo F., Montalto C., Denti P., Praz F., Sala A., Winkel M.G., Taramasso M., Tagliari A.P., Fam N.P. (2022). Management and Outcome of Failed Percutaneous Edge-to-Edge Mitral Valve Plasty: Insight From an International Registry. JACC Cardiovasc. Interv..

